# Effectiveness of Pharmacological Versus Non-pharmacological Methods for Managing Depression in Patients Undergoing Hospital Hemodialysis: A Narrative Review

**DOI:** 10.7759/cureus.94326

**Published:** 2025-10-11

**Authors:** Vignesh Vijayakumar

**Affiliations:** 1 Internal Medicine, Royal Gwent Hospital, Newport, GBR

**Keywords:** behavioral intervention, clinical effectiveness, cognitive behavioural therapy, depression, “efficacy”, end-stage kidney disease, haemodialysis (hd), non-pharmacological treatment, pharmacological treatment

## Abstract

Depression is one of the most common mental health issues prevalent in end-stage renal disease (ESRD) patients requiring hemodialysis, yet there is limited evidence on the best approach to treat depression in hemodialysis patients. A narrative review was conducted to examine the effectiveness of both pharmacological and non-pharmacological interventions, including antidepressant medications, dietary and herbal supplements, cognitive behavioral therapy (CBT), psychotherapy, acupressure, and exercise regimens to alleviate depressive symptoms and improve the quality of life as evaluated by various depression and quality of life screening tools and questionnaires.

A database search was conducted in the University of South Wales Library as well as the MEDLINE database, and relevant articles published in the English language and fitting the inclusion criteria, which mainly comprised articles assessing outcomes in terms of improvement in depression symptoms and quality of life, were selected for the purpose of this review. A total of 29 randomized controlled trials (RCT) were included in this review, of which eight studied pharmacological treatment methods and 21 studies examined non-pharmacological or psychosocial interventions to treat depression.

The pharmacological interventions include antidepressant medications and supplements, compared with a placebo or another active intervention. Sertraline, as a selective serotonin reuptake inhibitor (SSRI) in hemodialysis patients, has conflicting evidence base, whereas there is inconclusive evidence for the effectiveness of fluoxetine and citalopram. The role of supplements is promising in this population cohort and needs further research to justify their use in treating depression. The non-pharmacological methods include CBT, guided imagery, psychotherapy, endurance-resistance training, exercise training, mindfulness-based stress reduction, acupressure, tele-nursing, Benson relaxation technique, hope therapy, and internet-based intervention. CBT is the most widely studied intervention and proves to be effective against wait-listed patients, but does not prove to have a more beneficial effect when compared to psychotherapy. Various exercise regimens, including endurance-resistance training and intra-dialysis exercise, show good results in alleviating depressive symptoms, but further research is required in this field to establish these methods as a treatment option in the future. Other interventions such as hope therapy, internet-based measures, guided imagery, tele-nursing, Benson relaxation technique, and mindfulness-based stress reduction all have very limited evidence, although they have been trialed in studies.

This review combines both pharmacological and non-pharmacological treatment options and their effectiveness in treating depression in hemodialysis patients. Dietary and herbal supplements have low-grade evidence, whereas more research is required for SSRIs in treating depression. CBT is useful to improve depression scores and quality of life, along with psychotherapy. A standardized approach and follow-up of patients throughout the treatment and follow-up period, along with a greater sample size and assessment of outcomes through more standardized tools and questionnaires, would improve the quality of evidence collected, which will ultimately aid in establishing an evidence-based method of addressing an important health concern, that is, depression in hemodialysis patients.

## Introduction and background

Prevalence

The number of patients who are initiated on renal replacement therapy (RRT) for end-stage renal disease (ESRD), such as hemofiltration, hemodialysis, and peritoneal dialysis, has been steadily increasing over the years. Over 8,000 adult patients commenced RRT in 2017, which was 2.6% higher than that of 2016, and the prevalence of adults receiving RRT is also increasing, with the figures being 64,887 patients in 2017 [1]. The prevalence of chronic kidney disease (CKD) stages 3-5 is slated for an increase with the current trend, which proportionally increases the number of patients requiring RRT.

The prevalence of depression in ESRD patients undergoing dialysis was 39.3% and 22.8% when patients were examined through screening questionnaires and clinical interviews, respectively [2].

Background

Mental health-related disorders are common in patients undergoing dialysis. Depression is one of the major psychiatric problems associated with hemodialysis, and recent studies have shown that patients with CKD who are not on dialysis have rates of depression up to three times higher than those in the general population, and it is much higher in those patients requiring dialysis [2]. The impact of depression in chronic hemodialysis patients in terms of mortality or requiring hospitalization is significant, which is twice compared to ESRD patients without depression [3]. The risk of mortality in dialysis patients was found to be 1.5 times higher when patients exhibited symptoms of depression [4]. ESRD, as the name suggests, is the final stage of CKD, and these patients often have a poor quality of life [5].

There are various criteria to diagnose depression as listed in The Diagnostic and Statistical Manual of Mental Disorders (DSM-5) as well as in the International Classification of Diseases (Tenth Revision) [6,7]. The main symptoms of depression are sleep disturbance, including insomnia and hypersomnia, loss of weight, fatigue, feelings of worthlessness or guilt, and diminished ability to think, concentrate, or make decisions. There is a degree of overlap of a few of these symptoms, which often makes it difficult to differentiate features of depression from symptoms of ESRD. This problem can be overcome by means of conducting clinical interviews, of which the Structured Clinical Interview for DSM Disorders (SCID) [8], the Composite International Diagnostic Interview (CIDI) [9], and the Mini-International Neuropsychiatric Interview (MINI) [10] are considered gold-standard for this purpose.

Screening for depression is undertaken with tools such as structured interviews or questionnaires. Beck Depression Inventory (BDI), Patient Health Questionnaire (PHQ), Center for Epidemiologic Studies Depression Scale (CESD), and Quick Inventory of Depressive Symptomatology Self-Report (QIDS-SR) are the widely used screening tools for patients undergoing dialysis [5].

With regard to the treatment of depression in hemodialysis patients, there is no fixed treatment algorithm, and treatment is individualized to the patient’s symptoms and preferences. There are multiple options available, both pharmacological and non-pharmacological, for the treatment of depression in hemodialysis patients. Pharmacological options mainly consist of antidepressants and symptomatic treatment for anxiety and sexual dysfunction. Some of the commonly used antidepressants in this population cohort mainly consist of SSRIs such as sertraline, paroxetine, fluoxetine, and citalopram. Non-pharmacological methods include cognitive behavioral therapy (CBT) and alternative therapies such as counselling, social support, psychotherapy, music therapy, and exercise [5]. Non-pharmacological or psychosocial interventions show promising results in alleviating depressive symptoms and improving the quality of life, as established by some of the recent studies highlighted later in this article.

Etiology of depression in CKD

While there are various mechanisms resulting in depression in chronic kidney disease patients, the relationship between depression and CKD, like other illnesses, can be studied under behavioral and biological mechanisms. The burden of illness as a result of physical and psychological symptoms, self-care, functional impairment, and lack of social support leads to non-adherence, which in turn causes uncontrolled blood pressure, blood glucose, cholesterol levels, and fluid overload. A few biological factors, such as comorbidities, inflammatory response, hormonal abnormalities, and genetics, along with behavioral factors, cause increased cardiovascular events [11].

Inflammation levels are seen to be high in ESRD patients, which has a direct correlation with health outcomes and mortality rates [12]. There are various challenges that dialysis patients face, such as frequent visits to the dialysis center, restrictions on diet and various comorbidities, and its associated requirement of taking multiple medications. A study examining 210 chronic dialysis patients, among whom around 50% exhibited signs of depression, showed that family and personal issues, as well as financial burden, were significant factors leading to depression [13].

Research question

While there are multiple treatment modalities available for the treatment of depression in patients undergoing hemodialysis, there is limited evidence of the effectiveness of these interventions. Only one-fourth of dialysis patients diagnosed with depression receive treatment for the same [14].

Antidepressant treatment with selective serotonin reuptake inhibitors (SSRIs) is a commonly used method of treating depression. One of the studies shows that only 34.9% of patients undergoing hemodialysis and having a clinical diagnosis of depression were on antidepressant treatment [15].

Various aspects of mental and physical well-being are affected in hemodialysis patients in terms of quality of life, as well as due to polypharmacy by virtue of ESRD and other comorbidities associated with the nature of the disease. Most studies found promising findings with cognitive behavioral therapy; however, studies examining the combined efficacy of SSRIs and CBT are also very limited. Non-pharmacological interventions such as cognitive behavioral therapy and relaxation therapies have a positive effect in treating depression in hemodialysis patients, whereas the evidence surrounding acupressure and meditation is very weak and lacking.

Due to the conflicting views on the efficacy of pharmacological versus non-pharmacological interventions for treating depression and uncertainties arising from subjective screening of depressive symptoms, there are no established guidelines for the treatment of depression in the ESRD population cohort. This necessitates a review of the literature to assess the effectiveness of pharmacological versus non-pharmacological treatment methods for depression in hemodialysis patients and to provide an updated summary on potential benefits, disease-related outcomes, and quality of life of various treatment options currently used to treat depression in patients undergoing hemodialysis.

The research question aims to address the effectiveness of depression treatment in the adult population suffering from depressive symptoms and undergoing hemodialysis in the hospital.

**Table 1:** PICO elements PICO: Population, Intervention, Comparison, and Outcome; ESKD: end-stage kidney disease; CBT: cognitive behavioral therapy

PICO Element	Description
Population	Adults diagnosed with depression and ESKD requiring hospital hemodialysis
Intervention	Pharmacological (antidepressants) and non-pharmacological (CBT and alternative therapies)
Comparison	With placebo/pharmacological versus non-pharmacological
Outcome	Reduction in depressive symptoms and improvement in quality of life

Aims and objectives

Depression in hemodialysis patients is a major health concern, and there are no established guidelines for treatment in this population cohort [16]. Although several treatment options are available for treating depression in hemodialysis patients, the evidence of their effectiveness is limited. The main purpose of this review is to compare the effectiveness of pharmacological treatment options for depression with non-pharmacological or psychosocial interventions and to help establish an evidence-based approach to treating depression in hemodialysis patients.

Objectives of this review are to evaluate the effectiveness of pharmacological methods for treating depression in hemodialysis patients, to evaluate the effectiveness of non-pharmacological methods for treating depression in hemodialysis patients, to compare the effectiveness of pharmacological and non-pharmacological treatment methods for depression in hemodialysis patients and to provide an updated summary on the best interventions for treating depression in hemodialysis patients based on the review of relevant literature available.

Materials and methods

All relevant studies selected are in the English language and are primary source articles. Only studies that included adult patients and examined the outcome of either pharmacological or non-pharmacological intervention were selected. Since the effectiveness of these interventions is being assessed, the studies selected include only randomized controlled trials (RCTs), which are ranked high in the hierarchy of evidence for the nature of this study.

The inclusion criteria are all adults aged 19 years and above, ESRD patients requiring maintenance hospital hemodialysis, patients diagnosed with depression or showing clinical signs of depression at baseline, studies that investigate the treatment of depression in hemodialysis patients, studies that are published in the English language, and RCTs that evaluate the treatment of depression in patients requiring hemodialysis. The exclusion criteria are patients requiring hemodialysis due to acute kidney injury (AKI), patients requiring modalities of renal replacement therapy (RRT) other than hemodialysis, such as peritoneal dialysis, hemofiltration and transplantation, patients opting for conservative kidney management, patients diagnosed with mixed depressive disorder and other psychiatric illnesses and studies that are not classified as mentioned in the inclusion criteria, like posters, expert opinions and those available only in the abstract form.

Various databases were searched to identify relevant studies and articles from 1995 to June 2023. A preliminary search was conducted on Google Scholar and the University of South Wales library with the phrases “treatment of depression in hemodialysis” and “treatment of depression in dialysis,” and the relevant articles were gathered. A thorough search was conducted on the University of South Wales (USW) Library. MEDLINE database was also searched for relevant literature, and the studies were selected according to the eligibility criteria listed above. RCTs were selected due to the nature of the review involving intervention and effects.

The search strategy was devised using the keywords/MeSH (Medical Subject Headings) terms related to the available treatment modalities for depression in hemodialysis patients. The keywords for this narrative review were derived from the PICO elements addressing the research question as mentioned earlier. The Boolean Operator “AND” was used to combine keywords during the literature search. The Boolean Operator “OR” was used to search for synonyms of the same keyword. The keywords/MeSH terms used during the database search were depression, depressive disorder, dialysis, hemodialysis, end-stage renal disease, end-stage kidney disease, effectiveness, efficacy, outcome, treatment, intervention, therapy, pharmacologic, antidepressant, SSRI, non-pharmacologic, cognitive behavioral therapy, alternative therapy. 

A summary of the search strategy, along with the various keywords and MeSH terms used and the results in the MEDLINE database using eBSCO is illustrated in Table 2.

**Table 2:** Search strategy in MEDLINE Database

Keywords	Search results
"depression OR depressive disorder OR depressive symptoms OR major depressive disorder" AND "dialysis OR hemodialysis OR haemodialysis" AND "treatment OR intervention OR therapy"	189
		"depression OR depressive disorder OR depressive symptoms OR major depressive disorder" AND "dialysis OR hemodialysis OR haemodialysis" AND "treatment OR intervention OR therapy" AND "effectiveness OR efficacy OR effective"	343
				"depression OR depressive disorder OR depressive symptoms OR major depressive disorder" AND "end stage renal disease OR end stage renal failure OR chronic kidney disease or kidney failure" AND "treatment OR intervention OR therapy"	1961
						"depression OR depressive disorder OR depressive symptoms OR major depressive disorder" AND "dialysis OR hemodialysis OR haemodialysis" AND "pharmacological treatment OR pharmacological intervention OR pharmacological therapy"	27
"depression OR depressive disorder OR depressive symptoms OR major depressive disorder" AND "dialysis OR hemodialysis OR haemodialysis" AND "non-pharmacological interventions"	5						
								"depression OR depressive disorder OR depressive symptoms OR major depressive disorder" AND "dialysis OR hemodialysis OR haemodialysis" AND "cognitive behavioral therapy OR CBT OR cognitive behavioural therapy"	51
		"depression OR depressive disorder OR depressive symptoms OR major depressive disorder" AND "dialysis OR hemodialysis OR haemodialysis" AND "SSRI OR selective serotonin reuptake inhibitors OR antidepressants"	141						
				"depression OR depressive disorder OR depressive symptoms OR major depressive disorder" AND "dialysis OR hemodialysis OR haemodialysis" AND "alternative therapy"	12				

A summary of the search strategy, along with the various keywords and MeSH terms used and the results at the University of South Wales, is illustrated in Table 3.

**Table 3:** Search strategy in University of South Wales (USW) Library

Keywords	Search results
depression AND hemodialysis AND treatment	1154
depression AND hemodialysis AND treatment AND effectiveness	89
depression AND end stage renal disease AND treatment AND effectiveness	54
		depression AND end stage kidney disease AND treatment AND effectiveness	47
depression AND hemodialysis AND pharmacologic AND effectiveness	7		
		depression AND hemodialysis AND SSRI	4
depression AND hemodialysis AND anti depressant	99
depression AND hemodialysis AND non pharmacologic	34
depression AND hemodialysis AND non pharmacologic AND effectiveness	5
		depression AND hemodialysis AND alternative therapy	38
depression AND hemodialysis AND cognitive behavioral therapy	88

Search outcomes

The articles were screened for studies assessing the pharmacological treatment methods, mainly consisting of antidepressant medications, as well as non-pharmacological methods of CBT, alternative therapy, and exercise in the adult ESRD population requiring hemodialysis diagnosed with depression. All the results of the database search were screened by studying the titles and abstracts, and only those that are relevant to the literature review were chosen; the rest were eliminated from this study. Reference lists of the articles were examined for any missed studies and articles. Subsequently, the full text of the chosen articles was critically evaluated for inclusion. Final selection of the studies to be included in the review was based on Preferred Reporting Items for Systematic Review and Meta-analyses (PRISMA) guidelines.

A few studies were excluded as the inclusion criteria were for dialysis patients, including peritoneal dialysis and hemofiltration. Some articles were excluded as the population demographic included in the study was young adults, less than 19 years of age, and also three relevant articles were excluded due to unavailability of full-text articles. 

**Figure 1:**
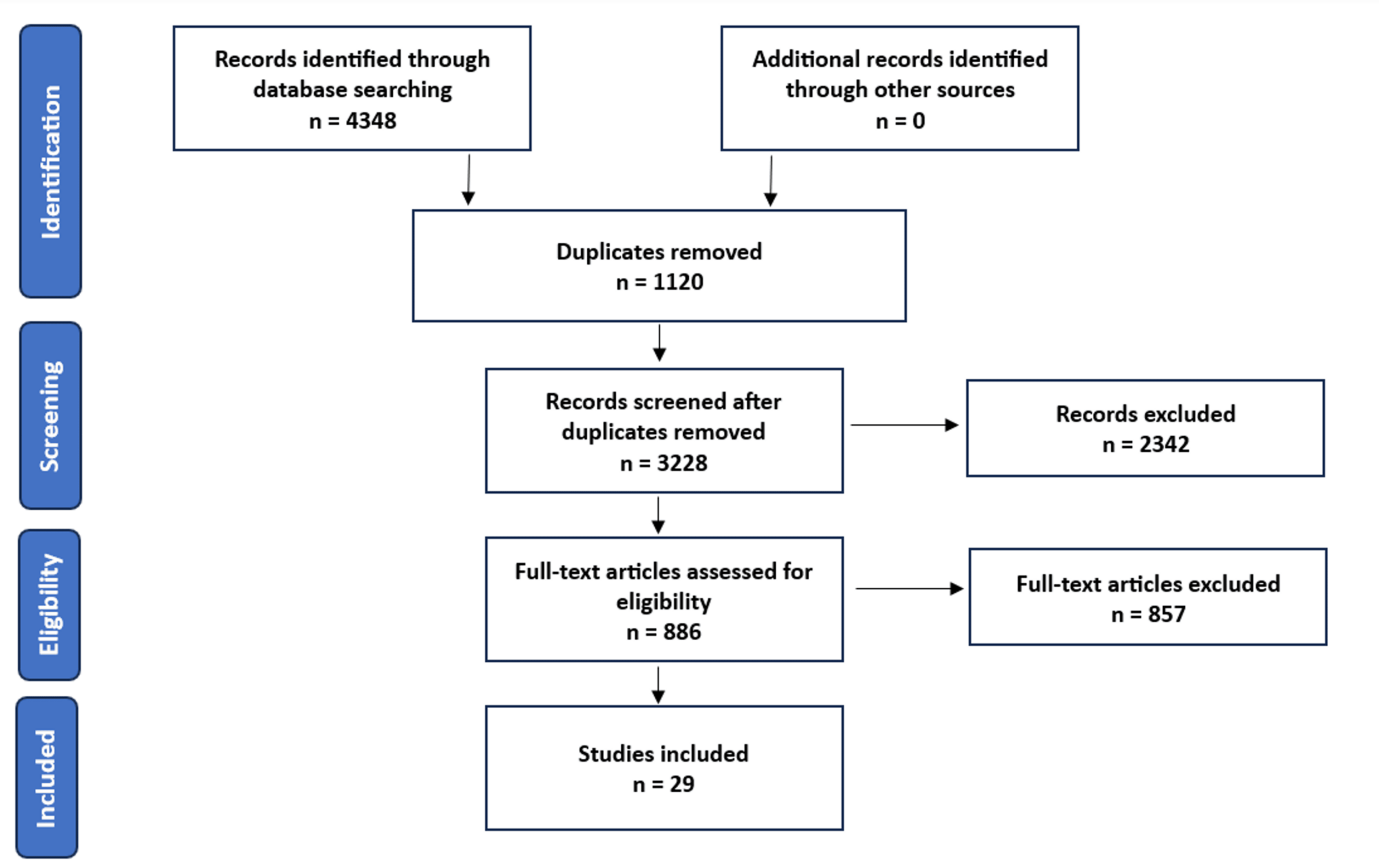
PRISMA Flow Diagram

The PRISMA flow diagram (Figure 1) depicts the selection of studies starting from searching the databases to the inclusion of relevant studies. Initial search of the University of South Wales Library using the keywords listed 1619 articles, and in the MEDLINE database revealed 2729 articles. De-duplication was done using the EndNote reference manager. The number of records after the duplicates were removed was 3228. Titles and abstracts of these articles were screened. Then, 2342 records were excluded due to not meeting the inclusion criteria, which included the study population being CKD, ESRD not initiated on hemodialysis, or studies that did not assess the effectiveness or efficacy of an intervention (pharmacological or non-pharmacological) against a placebo or another comparator. A total of 886 full-text articles were examined, out of which 857 were excluded as these studies did not assess outcomes in terms of improvement in quality of life, reduction in depressive symptoms. After exclusion of articles due to unavailability of full-text articles, finally, the total number of studies included in this systematic review is 29, all of which are RCTs.

Data collection and analysis

From the selected studies, study characteristics were assessed, and the data were synthesized was represented in a tabular column. The effectiveness of the various interventions or therapies was examined in terms of the improvement in quality of life (QOL) and reduction in the severity of depression. Various tools that were used to assess these outcomes in this study are the Hospital Anxiety and Depression Scale (HADS), Beck Depression Inventory-II (BDI-II), Montgomery-Asberg Depression Rating Scale (MADRS), Mini International Neuropsychiatric Interview (MINI), Hamilton Depression Rating Scale (HAM-D), Depression, Anxiety and Stress Scale - 21 Items (DASS-21), Zung Self-rating Depression Scale (Zung SDS), Kidney Disease-Related Quality of Life questionnaire (KD-QOL), Patient Health Questionnaire-9 (PHQ-9), Patient Satisfaction with Nursing Care Quality Questionnaire (PSNCQQ), and the World Health Organization Quality of Life (WHOQOL) [17-27].

Quality assessment

Critical appraisal is the process of examining the quality and reliability of evidence. Strength of evidence (SOE) was assessed using updated methodology from Grading of Recommendations Assessment, Development and Evaluation (GRADE) [28]. This method assesses the effectiveness of therapeutic interventions, and each outcome is scored in various domains by considering study limitations, reporting bias, consistency, and precision. SOE was classified as high, moderate, low, or insufficient. The Cochrane Risk of Bias (ROB) tool was used to assess RCTs. There are seven domains that this tool looks into, which are allocation concealment, random sequence generation, blinding of participants, blinding of outcomes, selective reporting, incomplete outcome data, and other sources [29]. Each of these is graded as high, low, or unclear risk of bias.

## Review

Study characteristics

Pharmacological Studies

From the database search, eight RCTs that satisfied our inclusion and exclusion criteria were identified for this study, of which four were conducted in Iran, two in the United States, and one each in England and China. Two studies employed HADS as the screening tool for depression, two studies used the BDI-II score for the same purpose, while the other studies examined depressive symptoms using other tools such as MADRS and MINI scale.

The qualitative data synthesized for RCTs assessing pharmacological methods of treating depression in adult hemodialysis patients are summarized in the table below (Table 4). 

**Table 4:** Study characteristics of studies assessing pharmacological treatment RCT: randomized controlled trial; HADS: Hospital Anxiety and Depression Scale; BDI: Beck Depression Inventory-II; MADRS: Montgomery–Asberg Depression Rating Scale; MINI: Mini International Neuropsychiatric Interview; HAM-D: Hamilton Depression Rating Scale; CBT: cognitive behavioral therapy

Study	Year	Study design	Study setting, country	Intervention	Inclusion criteria	Screening tool
Hosseini et al. [30]	2012	RCT	Hemodialysis center, Iran	Citalopram versus psychological training	HADS ≥ 8	HADS
							Freidli et al. [31]	2017	RCT	Renal centers, UK	Sertraline versus placebo	BDI-II score ≥16, MADRS score ≥18, MINI mild or moderate MDD	BDI-II, MADRS, MINI
														Blumenfield et al. [32]	1997	RCT	Dialysis centers, USA	Fluoxetine versus placebo	HAM-D score ≥16	HAM-D, BDI-II, MADRS
Haghighat et al. [33]	2019	RCT	Hemodialysis center, Iran	Synbiotic supplements versus probiotics versus placebo	HADS ≥ 8	HADS														
							Gharekhani et al. [34]	2014	RCT	Hemodialysis centers, Iran	Omega-3 fatty acid versus placebo	BDI-II score ≥16	BDI-II							
														Taraz et al. [35]	2013	RCT	Hemodialysis clinic, Iran	Sertraline versus placebo	BDI-II score ≥16	BDI-II
							Mehrotra et al. [36]	2019	RCT	Dialysis units, USA	Sertraline versus CBT	BDI-II score ≥15 confirmed by MINI	BDI-II, MINI							
Wang et al. [37]	2013	RCT	China	Radix Bupleuri supplements versus placebo	Non-psychotic and non-suicidal adults	MADRS							

A study conducted by Hosseini et al. examined 44 hemodialysis patients who were divided and allocated into treatment group (with citalopram) and comparator (psychological training) in a hemodialysis center in Iran [30]. HADS was used to compare the depression score before and after each intervention. The cut-off for inclusion was HADS ≥ 8. A multicenter, double-blind RCT in 30 hemodialysis patients in multiple centers in England was conducted that compared the effect of treatment with sertraline initially started at 50 mg/day and increased up to 100 mg/day at the end of the second and fourth months, with a matched placebo group [31]. BDI-II score ≥16, MADRS score ≥18, and MINI mild or moderate MDD were the inclusion criteria for this study. An RCT was conducted on 14 hemodialysis patients, out of which seven were treated with 20 mg fluoxetine / day and the other seven were in the placebo group [32]. This study was carried out in multiple dialysis centers in New York, USA. The effect of synbiotic supplements (15 g of prebiotics, 5 g of probiotic containing* Lactobacillus acidophilus* T16, *Bifidobacterium bifidum* BIA-6,* Bifidobacterium lactis* BIA-7, and *Bifidobacterium longum* BIA-8) and probiotic supplements (5 g of probiotics similar to synbiotics) and placebo group (20 g of maltodextrin) was examined, in which 16 patients in synbiotics group, 18 patients in probiotic group and 15 patients in placebo group were assessed over a period of 12 weeks in a hemodialysis center in Iran using the HADS score [33]. The effects of omega-3 fatty acids (180 mg eicosapentaenoic acid and 120 mg docosahexaenoic acid) in hemodialysis patients with depression versus the matched placebo group, who were given paraffin oil capsules, were studied [34]. The BDI score was used to assess the reduction in depressive symptoms among the two groups.

An RCT was conducted in hemodialysis patients in Iran, in which 25 depressed hemodialysis patients in an outpatient clinic in Iran were randomly allocated into the treatment group receiving sertraline and 25 patients to the placebo group, and were followed up over a period of 12 weeks [35]. The BDI-II score was used to assess whether the intervention proved to be effective. Another study was conducted in multiple dialysis units in the USA on 120 participants, 60 each in the treatment and control groups [36]. This study’s purpose was to examine the efficacy of sertraline in the treatment of depression in hemodialysis patients. BDI-II score, further confirmed by the MINI interview, was the inclusion criterion highlighted in this study. An RCT with 160 participants, out of which 80 took Radix Bupleuri herbal supplement and 80 were in the control group, was carried out in China [37]. MADRS was used as the screening tool for depression, and the RAND-36 item Health Survey (RAND-36) was used to assess quality of life.

Non-pharmacological Studies

A total of 21 studies, all of which were RCTs examining the effectiveness or efficacy of a non-pharmacological modality of treating depression in maintenance hemodialysis patients, were reviewed, and the data were synthesized for the purpose of this systematic review. In addition to the previously mentioned tools for assessment of depression and quality of life, additional ones employed include the Zung SDS, PHQ-9, DASS-21, PSNCQQ, and the WHOQOL questionnaire. The various interventions examined include CBT, psychotherapy, guided imagery, exercise training, mindfulness-based stress reduction therapy, Benson relaxation technique, tele-nursing, acupressure, hope therapy, internet-based intervention, CBI, and multifaceted educational intervention. The qualitative data synthesized for RCTs assessing non-pharmacological methods of treating depression in adult hemodialysis patients are summarized in Table 5.

**Table 5:** Study characteristics of studies assessing non-pharmacological treatment RCT: randomized controlled trial; BDI-II: Beck Depression Inventory-II; HAM-D: Hamilton Depression Rating Scale; MINI: Mini International Neuropsychiatric Interview; TAU: treatment as usual; HADS: Hospital Anxiety and Depression Scale; CBT: cognitive behavioral therapy; Zung SDS: Zung Self-Rating Depression Scale; MBSR: mindfulness-based stress reduction therapy; PHQ-9: Patient Health Questionnaire-9; DASS-21: Depression, Anxiety and Stress Scale-21 items; TEAS: transcutaneous electrical acupoint stimulation; CBI: cognitive behavioral intervention; KD-QOL: Kidney Disease-Related Quality of Life Questionnaire; PSNCQQ: Patient Satisfaction with Nursing Care Quality Questionnaire; WHOQOL-SF: World Health Organization Quality of Life-Short Form

Study	Year	Study design	Study setting and country	Intervention	Inclusion criteria	Screening tool
Cukor et al. [38]	2014	RCT	Dialysis units, USA	CBT vs. waiting list	BDI-II score ≥10	BDI-II; HAM-D
							Duarte et al. [39]	2009	RCT	Dialysis centers, Brazil	CBT vs. psychotherapy	BDI-II and MINI for screening depression	BDI-II; MINI
														Beizaee et al. [40]	2018	RCT	Hemodialysis center, Iran	Guided imagery vs. TAU	HADS score (no cut-off)	HADS
Al Saraireh et al. [41]	2018	RCT	Dialysis units, Jordan	CBT vs. psychoeducation	HAM-D score (no cut-off)	HAM-D														
							Frih et al. [42]	2017	RCT	Hospital, Tunisia	Endurance-resistance training vs. TAU	HADS score (no cut-off)	HADS							
														Kouidi et al. [43]	2010	RCT	Renal unit, Greece	Exercise training vs. control	No cut-off	BDI-II; HADS
							Lerma et al. [44]	2017	RCT	Hemodialysis units, Mexico	CBT vs. waiting list	BDI-II score 10 - 29	BDI-II							
Liao et al. [45]	2017	RCT	Hospital, China	Comprehensive nursing vs. normal care	Zung SDS (no cut-off)	Zung SDS							
							Thomas et al. [46]	2017	RCT	Hospital, Canada	MBSR vs. TAU	PHQ-9 score ≥6	PHQ-9
Heshmatifar et al. [47]	2015	RCT	Hemodialysis unit, Iran	Benson technique vs. TAU	BDI-II (no cut-off)	BDI-II							
							Kargar Jahromi et al. [48]	2014	RCT	Hemodialysis unit, Iran	Telenursing vs. TAU	DASS-21 (no cut-off)	DASS-21
Kalani et al. [49]	2019	RCT	Multiple hemodialysis centers, Iran	Acupressure vs. sham vs. TAU	BDI-II score ≥10	BDI-II							
Li et al. [50]	2020	RCT	Hospital, China	Home nursing visits vs. telephone follow-up	Zung SDS (no cut-off)	Zung SDS
							Rahimipour et al. [51]	2015	RCT	Multiple hospitals, Iran	Hope therapy vs. control	DASS-21 (no cut-off)	DASS-21
Tsay et al. [52]	2014	RCT	Multiple sites, Taiwan	Acupressure vs. TEAS versus control	BDI-II score ≥10	BDI-II							
							Hmwe et al. [53]	2015	RCT	Three hemodialysis centers	Acupressure vs. control	DASS-21 (no cut-off)	DASS-21
Nadort et al. [54]	2022	RCT	Nine hospitals, The Netherlands	Internet-based intervention vs. control	BDI-II score ≥10	BDI-II							
							van Vilsteren et al. [55]	2005	RCT	Dialysis center, The Netherlands	Pre-conditioning exercise program and counselling vs. normal care	RAND-36 score	RAND-36
Gonzalez-Flores et al. [56]	2023	RCT	Nephrology unit, Mexico	CBI plus resilience model vs. CBI	BDI and KD-QOL score	BDI; KD-QOL							
							Rigas et al. [57]	2022	RCT	Hemodialysis centers, multi-site, Canada	Brief mindfulness intervention vs. health enhancement program	PHQ-9 score ≥6	PHQ-9
Zhianfar et al. [58]	2020	RCT	Two hemodialysis wards, Iran	Multifaceted educational intervention vs. control	BDI, PSNCQQ, and WHOQOL-SF (no cut-off reported)	BDI; PSNCQQ WHOQOL-SF							

The study conducted in dialysis units in New York examined participants divided into a treatment group (receiving CBT) and a control group (wait-list period) [38]. BDI-II and HAM-D were used as self-reporting tools for depression, and KDQOL-SF was used to assess improvement in the quality of life of the participants in this study [38]. The effectiveness of CBT was tested in an RCT in 2009 wherein participants were randomized into two groups, one receiving CBT in group sessions for 90 minutes every week and the other group receiving individualized psychotherapy every week [39]. BDI, MINI, and the KDQOL-SF questionnaires were employed for this purpose, and participants were followed up over a period of nine months [39]. In 2018, an RCT was conducted on hemodialysis patients wherein 40 patients were randomly assigned to the guided intervention group (guided imagery) and TAU [40]. The levels of depression were assessed using the HADS score.

Another RCT was conducted across five dialysis units in Jordan to compare the effectiveness of CBT versus psychoeducation in treating depressive symptoms in hemodialysis patients [41]. A total of 65 participants were assigned randomly to each group, and the HAM-D was used to assess the effect of the intervention after a treatment duration of 12 weeks and a follow-up of 12 weeks. A single-blinded RCT was conducted on 50 participants in Tunisia, where 28 participants were assigned to the intervention group having one hour of exercise (endurance-resistance) training on non-dialysis days; 22 were allocated as sedentary controls [42]. The HADS score was used to assess the improvement of depressive symptoms in these patients. The efficacy of the exercise training program during the first two hours of dialysis session was tested during an RCT in a renal unit in Greece, where participants were allocated into an intervention group (exercise training) undergoing intra-dialysis exercises like warm-up, strengthening, cycling, and cool-down for about 60 to 90 minutes [43]. This group was compared with sedentary controls, and BDI-II and HADS were used to check the impact of the intervention.

In 2017, another RCT was conducted to study the effectiveness of CBT when compared to sedentary controls in hemodialysis units in Mexico [44]. Parameters used to assess the outcome included the BDI-II score, and quality of life were also assessed in this study. In another RCT study, 128 patients were examined to determine the effects of comprehensive nursing intervention on hemodialysis patients [45]. This included health education once or twice every week and CBT, as well as progressive relaxation therapy. This set of interventions was compared against routine conventional care. The Zung SDS was used to assess post-intervention outcome. Quality of life was assessed using the KDQOL-36. There was another RCT in 2017 involving 41 patients, wherein the intervention group received individual chair-side meditation during hemodialysis, compared with a control group [46]. PHQ-9 was used as the screening tool to measure improvement in depressive symptoms during this study. A RCT randomly allocated participants into a treatment group receiving the Benson relaxation technique (20-minute sessions) and into a control group (usual treatment) [47]. The BDI-II score was used to assess whether the participants had any improvement in reported depressive symptoms.

A double-blinded trial was done on 60 chronic hemodialysis patients, with 30 of them receiving routine conventional care, while 30 patients received telephone calls each for about 30 minutes post-dialysis sessions [48]. DASS-21 was used to evaluate the effectiveness of this intervention. An RCT was conducted in multiple hemodialysis sites in Iran [49]; 32 patients each were randomly allocated into three groups. The first was the intervention group, where hemodialysis patients received acupressure lasting for 20 minutes during the first two hours of dialysis, and received this intervention three times per week. The second group was the sham, where the patients received a similar intervention to acupressure, but the pressure points were different (1 cm from the acupressure points), and the third group was the control group (TAU). The BDI-II score was used to assess the effectiveness of this intervention [49].

The impact of home nursing visits on hemodialysis patients was studied, where patients were divided randomly into two groups of 36 each, one receiving the intervention and the other group as the control [50]. Zung SDS was used as the tool to screen depressive symptoms, and quality of life was assessed using the KDQOL-36 tool. This study was conducted in a hospital in China. In 2015, another RCT was conducted in multiple hospital sites in Iran where 25 hemodialysis patients each were assigned to treatment groups (receiving 60 to 90 minutes of hope therapy during the first two hours of dialysis) and to the placebo (involving listening sessions) [51]. DASS-21 was employed as the screening scale to assess the depressive symptoms and the effect of the intervention. A study on 108 hemodialysis patients was held to measure the effectiveness of acupressure and transcutaneous electrical acupoint stimulation (TEAS) on hemodialysis patients when compared to a control group [52].

An RCT was carried out to assess whether acupressure can be used as a treatment option for hemodialysis patients was carried out across three dialysis centers [53]. DASS-21 was used as the tool to assess depression. In 2022, an RCT was conducted where a total of 190 hemodialysis patients were randomly enrolled into the study into the intervention group involving internet-based problem-solving training consisting of five modules with information, examples, and assignments for a period of 12 weeks. The control group involved usual care [54]. This study was carried out across multiple dialysis units in hospitals across the Netherlands. The BDI-II score was used to assess the effectiveness of the intervention.

In 2005, an RCT was conducted assessing the effectiveness of a pre-dialysis strength training program and a cycling (during dialysis) program on patients undergoing hemodialysis [55]. Patients in the treatment group underwent exercise training and counselling, each lasting for about 20 to 30 minutes twice or thrice every week. The Dutch version of RAND-36 was used to measure the patient’s quality of life [55]. Another RCT was conducted in Mexico in the nephrology department of a hospital in Mexico that studied the effect of cognitive behavioral intervention + resilience (CBI + R) versus cognitive behavioral intervention (CBI) using the BDI score and the KD-QOL score [56]. Resilience was evaluated using the Mexican Resilience Scale.

An RCT was carried out to assess whether brief mindfulness interventions are better in alleviating depression symptoms in adult hemodialysis patients when compared to a health education program (HEP) [57]. The treatment group included mindful eating, body scanning, and guided breath meditation of 20-minute sessions twice a week, and the control group involved educational and activity-based sessions for 20-minute sessions twice a week during the treatment period of eight weeks, which was followed by a six-month follow-up. PHQ-9 was used to assess depression at baseline, after the treatment, and follow-up period. Another RCT was conducted in two hemodialysis wards in Iran that compared the effectiveness of a multifaceted educational intervention versus control (receiving regular care) [58]. The patients in the intervention group received multiple interventions, including educational video tracks, CBT sessions, and telephone-based peer support. BDI-SF, PSNCQQ, and WHOQOL-SF were used to assess the depressive score and quality of life.

Study outcomes

The key findings of each study including the number of participants, duration of treatment and follow up, the intervention of the study and the control or comparator against which the study population was trialed, along with the main findings of the study in assessing change in depression or quality of life post-intervention or post-follow up was collected and the evidence was synthesized in a tabular format. This section gives data on the 29 included studies and the findings on whether the proposed intervention has had an impact on depressive symptoms or on the quality of life, the two primary outcomes being assessed in this review. The P-value is a statistical measure that is used for testing the hypothesis. The lower the p-value, the higher the statistical significance.

Pharmacological Studies' Outcomes

A summary of the outcome of the included studies assessing pharmacological treatment options against a placebo or against another intervention is provided in Table 6.

**Table 6:** Study outcome - pharmacologic treatment T: treatment; C: control; F/U: follow-up; BT: cognitive behavioral therapy; HADS: Hospital Anxiety and Depression Scale; BDI-II: Beck Depression Inventory; MADRS: Montgomery-Asberg Depression Rating Scale

Study	Sample Size (N)	Duration of Treatment (Rx) Follow-up (F/U)	Treatment/ Intervention	Control/ Comparator	Findings/ Outcome		Quality of Evidence
								Hosseini et al. [30]	44	Rx = 3 months; F/U = 3 months	Citalopram 20 mg/day	Psychological training, six 1-hour sessions	T: HADS (P=0.002); C: HADS (P=0.045)		Poor
Freidli et al. [31]	30	Rx = 6 months; F/U = 6 months	sertraline 50 mg/day (up to 100 mg/day at 2nd and 4th months)	Placebo	T: MADRS -14.5 (95% CI, -20.2 to -8.8); C: -14.9 (95% CI, -18.4 to -11.5)		Fair								
																Blumenfield et al. [32]	14	Rx = 8 weeks; F/U = 8 weeks	Fluoxetine 20 mg /day	Placebo	BDI-II, T: -9.57; C: placebo -8.8 (P=0.91), HAM-D: T: -9.00; C: -7.5 (P=0.72)		Poor
								Haghighat et al. [33]	49	Rx = 12 weeks; F/U = 12 weeks	Synbiotic supplement, probiotic supplement	Probiotic supplement, placebo	Synbiotic versus probiotic, P=0.011, synbiotic versus placebo, P<0.001		Good								
Gharekhani et al. [34]	54	Rx = 4 months; F/U = 4 months	Omega-3 fatty acids 1800 mg / day	Placebo	BDI-II (SD), T: 13.44 (5.66); C: 20.33 (7.56)		Poor																
																Taraz et al. [35]	50	Rx = 12 weeks; F/U = 12 weeks	Sertraline 50 mg / day for first 2 weeks, then 100 mg /day	Placebo	BDI-II score (SD) from baseline to 12 weeks, T: -11.3 (5.8); C: -0.5 (5)		Fair
								Mehrotra et al. [36]	120	Rx = 12 weeks; F/U = 12 weeks	Sertraline 200 mg / day	CBT: Ten 1-hour sessions	BDI-II scores, T: 14.1 (95% CI, 11.2 to 17.0); C: 18.7 (95% CI, 15.2 to 22.2)		Fair
Wang et al. [37]	160	3 months	Radix Bupleuri supplement (herbal)	Placebo	MADRS score, mean (SD); T: 13.32 (8.25); C: 16.73 (9.46)		Poor								

Patients on citalopram reported a decrease in their depression score (P = 0.001) and the total HADS score (P=0.002). On the other hand, patients undergoing psychological training, which included education about the disease, problem-solving, stress management, and muscle-relaxation techniques, reported a decrease in depression scores (P = 0.04) and total HADS (P = 0.045). Both these interventions yielded similar results in improving symptoms of depression and thus concluded that both reduced depressive symptoms in the hemodialysis population cohort, but the quality of the evidence overall is poor, and there is no difference between the two groups [30].

The study by Freidi et al. found no significant difference in reducing depression when comparing patients on antidepressant treatment (sertraline) with the placebo group, although the MADRS within-groups recorded a decrease in both the study groups [31]. Fluoxetine, as an antidepressant medication, is efficacious in hemodialysis patients when compared to the matched placebo group, according to the study by Blumenfield et al. However, there is no significant difference in the reduction of depressive symptoms as measured by the tools of depression mentioned in the study between the groups [32].

Patients on synbiotic supplements experienced a reduction in HADS score compared to probiotic and placebo counterparts. The mean difference in HADS scores from baseline is as follows: synbiotic: 22.24 (95% CI, 23.29 to 21.38), probiotic: 21.28 (95% CI, 22.05 to 20.53), placebo: 0.20 (95% CI, 20.43 to 0.76). Thus, synbiotic supplementation is an effective method to treat depression in hemodialysis patients [33]. Omega-3 supplements have proven to reduce BDI score after a four-month period of follow-up compared to the placebo group [34]. There was a significant decrease (P<0.001) in the treatment group versus no significant change in the placebo group. However, the quality of evidence is poor to quantify the effect of omega-3 supplements. Compared with the placebo group, 47.5% of patients reported an improvement in their depressive symptoms according to the study by Taraz et al. [35]. This provides a fair level of evidence that sertraline can be used as an effective strategy to treat depression in this population cohort.

The study conducted by Mehrotra et al. assessing the effect of an antidepressant (sertraline) versus CBT shows that after 12 weeks of treatment, depression scores seemed to be slightly better with treatment group compared to the CBT group, although it is to be noted that both the groups improved significantly, which is shown by the effect estimate: 22.9 (95% CI, 26.7 to 0.8) [36]. The findings of the study by Wang et al. suggest that patients in the treatment group, that is, taking the herbal supplement, had a greater reduction in MADRS scores compared with the placebo group (P=0.02). Similarly, the quality of life (measured by RAND-36) was also reported to be improved in the treatment group against the placebo group (P=0.04) [37].

Non-pharmacological methods studies' outcomes

A summary of the outcome of the included studies assessing non-pharmacological treatment options against a placebo or against another intervention is provided in Table 7.

**Table 7:** Study outcome - nonpharmacologic treatment T: treatment; C: control; F/U: follow-up; CBT: cognitive behavioral therapy; BDI-II: Beck Depression Inventory; TAU: treatment as usual; HEP: health education program; HADS: Hospital Anxiety and Depression Scale; PHQ-9: Patient Health Questionnaire-9; CBI: cognitive behavioral intervention

Study	Sample size (N)	Duration of treatment (Rx); F/U	Treatment/ Intervention	Control/ Comparator	Findings / Outcome		Quality of Evidence
								Cukor et al. [38]	59	Rx = 3 months; F/U = 6 months	CBT	Waitlist	BDI-II mean score (SD), T: -11.7 (1.5), P<0.001; C: -4.8 (1.4), P<0.001; HAM-D score, T versus C, P<0.001		Fair
Duarte et al. [39]	90	Rx = 3 months; F/U = 9 months	CBT (group sessions of 90 mins every week)	Psychotherapy (individual) once per week	BDI-II score (SD), T: 10.8 (8.8); C: 17.6 (11.2), P=0.002		Fair								
																Beizaee et al. [40]	80	Rx = 4 weeks; F/U = 4 weeks	Guided imagery (three sessions per week)	TAU	HADS score post follow-up, T: 10.02 (SD, 2.58); C: 11.65 (SD, 2.33)		Fair
								Al Saraireh et al. [41]	130	Rx = 12 weeks; F/U = 12 weeks	CBT (seven 1-hour sessions)	Psychoeducation (seven 1-hour sessions)	Post-intervention HAM-D score (SD), T: 15.0 (5.5); C: 11.1 (2.3)		Poor								
Frih et al. [42]	50	Rx = 4 months; F/U = 4 months	Endurance-resistance exercise training (1-hour sessions)	Sedentary control	HADS score improved in the intervention group (P<0.01)		Poor																
																Kouidi et al. [43]	50	Rx = 1 year; F/U = 1 year	Intra-dialysis exercise training program (three sessions per week)	Sedentary control	BDI-II score, P<0.001; HADS score, P<0.001		Poor
								Lerma et al. [44]	60	Rx = 5 weeks; F/U = 9 weeks	(group sessions)	Waiting list	BDI-II score post-follow-up, T: 7.1 (SD, 7.2); C: 14.7 (SD, 9.7), P=0.003		Fair
Liao et al. [45]	128	Rx = during admission; F/U = 3 months	Comprehensive nursing	Conventional care	Post intervention SDS score, T: 51.02 (SD, 20.59); C: 60.06 (SD, 28.91), P=0.04		Poor								
																Thomas et al. [46]	41	Rx = 8 weeks; F/U = 8 weeks	Mindfulness-based stress reduction (3 times per week)	TAU	PHQ-9 score, T: -3.0 (SD, 3.9); C: 2.0 (SD, 4.7), P=0.45		Fair
								Heshmatifar et al. [47]	70	Rx = 1 month; F/U = 1 month	Benson relaxation technique (20-min sessions)	TAU	T versus C: P=0.01		Poor								
Kargar Jahromi et al. [48]	60	Rx = not reported; F/U = 1 month	Telenursing (30-min sessions)	TAU	Post-intervention mean (SD), T: 8.96 (1.17); C: 16.20 (1.60), P=0.05		Poor																
																Kalani et al. [49]	96	Rx = 4 weeks; F/U = 4 weeks	Acupressure (20-min sessions) thrice a week	Sham: different location pressure; Control: TAU	Post-intervention BDI-II, acupressure: 20.6; sham: 25.5; C: 24.9		Fair
								Li et al. [50]	72	Rx = 6 months; F/U = 6 months	Home nursing visits	Telephone follow-up	Post-intervention SDS score, T: 36.48 (SD, 5.06); C: 48.80 (SD, 5.27)		Poor								
Rahimipour et al. [51]	50	Rx = 8 weeks; F/U = 12 weeks	Hope therapy (1 session per week)	Control: listening sessions (1 session per week)	Post-intervention: t=12.75; P<0.001; post-follow up: T=13.83; P<0.001)		Poor																
																Tsay et al. [52]	108	Rx = 4 weeks; F/U = 4 weeks	Acupressure (15-min sessions) thrice a week	Control	Acupressure (P=0.009) and TEAS (P=0.008) compared to control		Poor
								Hmwe et al. [53]	108	Rx = 4 weeks; F/U = 4 weeks	Acupressure (15-min) thrice a week	Control (routine care)	Post-intervention score for acupressure better than control		Fair								
Nadort et al. [54]	190	Rx = 12 weeks; F/U = not mentioned	Individual guided IPST	Control (normal care)	Post-intervention between groups (mean difference − 0.1, 95% CI -3.0; 2.7, P = 0.94)		Fair																
																van Vilsteren et al. [55]	96	Rx = 12 weeks; F/U = not mentioned	Pre-conditioning exercise program (20-30-min sessions)	Control (routine care)	Post-intervention quality of life improved compared to control group		Poor
								Gonzalez-Flores et al. [56]	53	Rx = 8 weeks; F/U = 4 weeks	CBI + resilience	CBI	Post-intervention mean (SD), T: 4.93 (3.91); C: 7.44 (3.38), P<0.01		Fair								
Rigas et al. [57]	55	Rx = 8 weeks; F/U = 6 months	Brief mindfulness intervention (20 minutes) twice a week	HEP (20 minutes) twice a week	Post-follow-up reduction in depression in both groups		Fair																
																Zhianfar et al. [58]	70	Rx = 1 month; F/U = 3 months	Multifaceted educational intervention	Control	Post-intervention mean (SD) depression, T: 6.24(3.59); C: 7.97(5.23); QOL, T: 91.24(1.38); C: 80.48(21.03)		Fair

According to Cukor et al., there was a greater degree of reduction in BDI-II (self-reported) scores in the treatment group (P=0.03) undergoing CBT and HAM-D Scale (P< 0.001). Another outcome that was measured was the quality of life using the KD-QOL SF (P=0.04), proving that the CBT group had a better quality of life and reduction in depressive symptoms [38]. The study by Duarte et al. found that there was a remarkable difference in treating depression as well as improved quality of life in the treatment group when compared to the comparator group receiving psychotherapy [39]. The study by Beizaee et al. gave a fair quality of evidence that guided imagery is beneficial in lowering the levels of depression as assessed by HADS score (P=0.030 in the intervention group, P=0.001 in the control group) when compared with the control group and could be considered as an effective treatment strategy to treat depression in hemodialysis patients [40].

The study conducted by Al Saraireh et al. concluded that CBT and psychoeducation groups were both effective methods to reduce depression in hemodialysis patients. Psychoeducation proved to have the upper hand in this study (P<0.01) over CBT [41]. The results of the study conducted by Frih et al. show that in the intervention group undergoing endurance-resistance training, the HADS score reported by patients had a significant improvement when compared to the control group [42]. The study by Kouidi et al. shows that patients on exercise training had a greater reduction in the levels of depression as assessed by the BDI-II and HADS score, and the effect was more noticeable in those patients who were severely depressed [43]. 

The results of the study conducted by Lerma et al. satisfactorily provide evidence that group sessions of CBT greatly reduce depression in chronic hemodialysis patients and also improve quality of life. In the treatment group, reduction in scores was P<0.001 compared to P=0.87 in the sedentary control group [44]. The study conducted by Liao et al. shows that patients undergoing comprehensive nursing care had significantly higher levels of reduction in depression as measured by the Zung SDS. Another finding of this study was that there was a significant improvement in quality of life as reported by the KDQOL-36 instrument [45].

Mindfulness meditation has been shown to be feasible as a treatment strategy for depression in hemodialysis patients; however, the limitation is that it did not find any significant effects on reducing depressive symptoms [46]. The study by Heshmatifar et al. shows that in the intervention group, the scores as assessed by BDI-II reduced significantly compared to the control group receiving usual care, and hence the Benson relaxation technique can be used as an effective treatment strategy for treating depression in hemodialysis patients [47]. Another study exploring non-pharmacological interventions was conducted by Kargar Jahromi et al. which demonstrates that in the group receiving the intervention, that is, telephone calls after dialysis sessions, there seemed to be a significant improvement in depression as assessed by the DASS-21 scoring criteria and thus gives promising evidence that this can be used as an effective method of treating depression in this population cohort [48].

There was a noticeable difference in the mean scores in the intervention group (receiving acupressure) when compared to the sham group and control (P=0.001), but no significant difference between the placebo (sham) and the control group (P=0.22) as per the findings by Kalani et al. [49]. When examining the effect of the intervention in the study conducted by Li et al., it is seen that both the treatment and well as the control groups experienced a decline in Zung SDS scores (P<0.001). Another important finding of this study is that the quality of life improved in both these groups, although the improvement is slightly higher in the intervention group [50]. The study conducted by Rahimipour et al. shows that the placebo group did not witness any improvement in their depressive symptoms as assessed by the DASS-21 score. However, the hemodialysis patients receiving active intervention (hope therapy) experienced an improvement in their mean scores, implying that hope therapy is a promising intervention to treat depression in chronic hemodialysis patients [51].

Tsay et al. conducted a study to examine the effects of acupressure and TEAS, which demonstrated that both were effective methods to reduce depression in the study population, but the control group did not yield similar results. Although other parameters were also measured in this study, including fatigue scale and quality of sleep, the study concluded that acupressure and TEAS both are effective strategies and have shown similar results in treating depression in maintenance hemodialysis patients suffering from depressive symptoms [52]. The findings of the study conducted by Hmwe et al. show that acupressure is an effective treatment modality for depression in dialysis patients, as there was a significant reduction in depression in patients undergoing acupressure in 15-minute sessions three or four times a week, as against the control group receiving routine conventional care [53]. There was no difference between the two groups studied in the RCT conducted by Nadort et al. Guided internet-based problem technique for dialysis patients provided similar results when compared to the routine as-usual-care in the participants being studied. The conclusion drawn from this study is that internet-based problem-solving training is not beneficial in reducing depression symptoms and cannot be employed as an alternative to the treatment of depression [54].

The findings of the study done by van Vilsteren et al. indicate that the intervention of pre-dialysis exercise training and counselling seems to improve the quality of life as assessed by the RAND-36 score, although the duration of intervention of 12 weeks was highlighted to be inadequate to quantify the scale of effect of the intervention on dialysis patients [55]. The results of the study by Gonzalez-Flores et al. were promising, as they showed that the treatment group had greater levels of improvement in their somatic depressive symptoms when compared with the control group (receiving only CBI), although the control group also exhibited improvement in depression and quality of life overall [56]. Although the treatment and control groups both exhibited a reduction in depression as assessed by the PHQ-9, there was no remarkable difference between the two groups [57].

The study performed by Zhianfar et al. shows that there was a significant change in the self-reported depression symptoms (P=0.001), Nursing Care Satisfaction score (P=0.001), and quality of life score (P=0.001). This yields promising findings that multifaceted interventions can be an effective way and a cost-effective method to improve depressive symptoms as well as the quality of life in hemodialysis patients [58].

Analysis of pharmacological methods

Three studies looked at the effectiveness of antidepressant medication (SSRIs) and compared them against a placebo (Blumenfield et al., Freidli et al., Taraz et al.) [31,32,35]. Fluoxetine did not come across as an effective treatment option, as there was no significant difference between the treatment and placebo groups. There is a conflicting result in the efficacy of sertraline, as one study (Taraz et al.) shows there is significant improvement in the treatment group, whereas another study (Blumenfield et al.) does not identify any improvement in depression score in the treatment group [32,35].

The role of dietary supplements - omega-3 fatty acids, synbiotic supplements, and Radix Bupleuri supplement was assessed in the studies conducted by Gharekhani et al., Haghighat et al., and Wang et al., respectively [33, 34, 37]. All three studies suggest that dietary supplements have a role in the treatment of depression, as these interventions have shown a reduction in depression scores after the period of treatment, as well as follow-up.

Two studies examined the effectiveness of SSRIs against another active comparator (Mehrotra et al. and Hosseini et al.) [30, 36]. The former study shows a significant reduction in depression scores in the treatment group receiving sertraline as well as in the control group (receiving CBT); however, the difference is more predominant in the treatment group. The latter study does not prove that citalopram is better than psychological training, as both these groups exhibited a lowering in their depressive symptoms.

Analysis of non-pharmacological methods

Multiple studies examined non-pharmacological methods of treating depression, which was more than double the number of studies that utilized pharmacological options. CBT was assessed in four of these studies, out of which two compared against a wait-list and the other two against psycho-education/psychotherapy. CBT was proven to greatly improve depressive symptoms as well as the quality of life when compared against waiting-list patients (Cukor et al. and Lerma et al.) [38,44]. Small group CBT was more effective than individual psychotherapy for decreasing the severity of depression in hemodialysis patients (Duarte et al.) [39]. CBT did not yield better results in treating depression when compared to psycho-education (PSE) as assessed by the study conducted by Al Saraireh et al. [41]. Both these groups exhibited improvement in depression scores and thus provided no conclusive evidence.

Three studies assessed the role of some type of exercise training in improving depression and overall quality of life. Endurance resistance training, exercise training, and pre-conditioning exercise training were assessed against normal or routine care in the studies conducted by Frih et al., Kouidi et al., and van Vilsteren et al., respectively [42,43,55]. The former two studies proved that exercise training yields satisfactory results in improving the outcome measured when put against the control group receiving routine care [42,43]. Pre-dialysis exercise training also proved to be beneficial in improving the quality of life (van Vilsteren et al.) [55]. The combined results of these three studies give promising insight into the use of exercise therapy as an effective modality for treating depression in hemodialysis patients.

Three studies looked at acupressure as a treatment option in hemodialysis patients. Acupressure versus sham versus control, acupressure versus transcutaneous electrical stimulation (TEAS) versus control, and acupressure versus control were examined by Kalani et al., Tsay et al., and Hmwe et al. [49,52,53]. These three studies collectively show that acupressure is more effective than the control or sham group in alleviating depressive symptoms and the quality of life; however, the beneficial effects of acupressure against TEAS do not have convincing evidence base.

Three studies explore nursing as an option to treat depressive symptoms. Liao et al., Kargar Jahromi et al., and Li et al. tested the effectiveness of comprehensive nursing care, tele-nursing, and home nursing visits, respectively [45,48,50]. The findings of these three studies provide insufficient evidence for the efficacy of nursing therapy. However, there was a noticeable reduction in depression (as measured by Zung SDS score) after treatment and also between the treatment and control groups. Mindfulness-based intervention was examined by two studies conducted by Rigas et al. and Thomas et al., wherein brief mindfulness intervention and mindfulness-based stress reduction (MBSR) were assessed against a control group [57,46]. These studies concluded that although this is a feasible option to be considered, there was a reduction in both the treatment and control groups, questioning the actual benefit of mindfulness-based therapies.

Guided imagery was assessed by Beizaee et al., who concluded that this intervention gives good quality evidence that it has a beneficial role in treating depression in hemodialysis patients [40]. Other modalities included in this review are hope therapy, which has poor evidence to show significant improvement, cognitive behavioral intervention with the Resilience model, and multifaceted educational intervention, which show that these are innovative and effective methods to address the research question in hemodialysis patients [52,55].

Recommendations

There is not an adequate number of studies examining individual interventions or treatment options against a control group or another intervention that would enable data to be quantified. Standardized study methodology and measures of outcomes will allow for a more thorough comparison of the different types of intervention available for treating depression in ESRD patients on maintenance hemodialysis, particularly because this is a growing burden on this population cohort and an important health concern that needs optimization of treatment.

More research on this topic is required in order to improve health outcomes as well as quality of life in hemodialysis patients. The majority of the studies conducted utilize SSRIs or CBT for treating depression; however, future studies should consider exploring more cost-efficient methods and utilizing the latest technological advancements, such as internet-based therapy and a combination of multi-dimensional approaches in order to improve patient outcomes.

Comparing an active intervention against another intervention, instead of a control group, widens the scope of understanding the effectiveness of both pharmacological and non-pharmacological treatment options. Outcomes being measured have been found to be largely subjective due to the usage of multiple depression scoring scales and questionnaires. The most common outcomes of reduction in depressive symptoms, as well as quality of life, need to be assessed using a more standardized approach and by ensuring that participants are followed up through the entire period of study, as many of the studies included in the review have lost a significant number of participants in the follow-up period, thus reducing the credibility of the studies. Future studies should focus on a more systematic and standardized methodology for recruiting participants and randomization in the treatment and control groups, and also focus on participant retention and examine outcomes after the intended treatment period and follow-up period. More sample size and a multi-centered approach with standardized use of questionnaires will help to gather more reliable evidence, and a standardized protocol can be laid out for treating depression in hemodialysis patients, which will greatly aid in addressing the growing burden of depression in these patients.

Strengths and limitations

The strength of this study is that a thorough search was conducted during the database search, as mentioned previously, and full-text articles were examined thoroughly based on the eligibility criteria. This review examines both pharmacological and non-pharmacological interventions and compiles data relevant to this study until June 2023. On the other hand, the database search only accounted for articles in the English language, and this could have potentially missed out on important studies that fit the criteria published in other languages. Another limitation is that a few studies were not included in this review due to limited access to the full-text article.

There are no standardized outcomes that are being assessed due to the usage of multiple screening tools and questionnaires. Another factor that hinders the reliability of the results is that the outcomes being measured are mostly subjective and can differ among patients. The short duration of studies and follow-up, and small sample size limit the reproducibility of these findings to the larger population. The tools used to assess quality of life and screening are not standardized, leading to an inability to standardize outcome assessment. 

## Conclusions

This literature review analyzed 29 RCTs on depression treatment in hemodialysis patients; 8 pharmacological and 21 non-pharmacological. Evidence for SSRIs (sertraline, fluoxetine, citalopram) remains inconsistent: while some studies show potential benefit, others highlight limited efficacy and concerns about polypharmacy. In contrast, non-pharmacological approaches provide stronger and more consistent results. CBT regularly reduces depressive symptoms compared to waitlisted patients, though it is not superior to psychotherapy; both approaches are effective. Exercise interventions (pre-dialysis, intra-dialysis, or training programs) also demonstrate meaningful improvements, while acupressure appears promising as an adjunct to CBT. Dietary supplements such as omega-3 fatty acids and herbal options show moderate evidence of benefit.

Across studies, limitations include small sample sizes, short treatment and follow-up durations, and inconsistent methodologies, making it difficult to generalize findings. Overall, non-pharmacological strategies, particularly CBT, exercise, and dietary supplements, emerge as the most reliable means of improving depression and quality of life in this patient group. Future research should aim for higher-quality trials, explore technology-driven approaches such as telemedicine and online therapy, and address psychosocial aspects such as financial and emotional burden, in addition to symptom management.
